# ANTI-ASIAN RACISM AMONG OLDER KOREAN AMERICANS DURING THE COVID-19 PANDEMIC

**DOI:** 10.1093/geroni/igad104.1864

**Published:** 2023-12-21

**Authors:** Soonhyung Kwon

**Affiliations:** Rutgers, the State University of New Jersey, Savoy, Illinois, United States

## Abstract

**Background:**

Hate crimes in the U.S. targeting adults of Asian descent have sharply increased during the COVID-19 pandemic. Korean Americans experience the highest level of everyday racism (37.5%), followed by Chinese (15.2%) and Vietnamese during the pandemic (Jung et al., 2021). Moreover, perceived discrimination in older Korean Americans is significantly related to a greater risk for poor mental health (Chau et al., 2018). However, older Korean Americans were likely to experience barriers to mental healthcare services. With this concern in mind, this study explored how older Korean Americans perceived discrimination related to ethnicity/race and coped with adverse mental health during the pandemic.

**Methods:**

Our study used the thematic analysis of textual data to reconstruct and categorize, engaging in six steps: 1) familiarizing the qualitative data, 2) generating initial codes, 3) searching for themes, 4) reviewing themes, 5) defining and naming themes, and 6) producing a final report.

**Results:**

This study identified that older Korean Americans (N=18) experienced direct and vicarious racism during the pandemic but felt barriers (limited English proficiency and a lack of accessible resources) to mental healthcare services. Active religious activities ameliorated discrimination-related stress. Some participants tried to solve the stress through wrong behaviors that criticized Chinese Americans. As a result, barriers to mental healthcare were likely to cause secondary discrimination and prejudice against Chinese Americans.

**Discussion:**

To ameliorate adverse mental health and prevent secondary discrimination, it is essential to identify well-accepted, well-timed, sustainable, and cost-effective therapeutic strategies to alleviate the adverse mental health associated with discrimination-related stress.

